# Intracutaneous suture versus transcutaneous skin stapling for closure of midline or horizontal skin incision in elective abdominal surgery and their outcome on superficial surgical site infections–INTRANS: study protocol for a randomized controlled trial

**DOI:** 10.1186/1745-6215-15-25

**Published:** 2014-01-16

**Authors:** Katja Maschuw, Christine Heinz, Elisabeth Maurer, Alexander Reuss, Carmen Schade-Brittinger, Detlef Klaus Bartsch

**Affiliations:** 1 Department of Visceral, Thoracic and Vascular Surgery, University Hospital Giessen and Marburg GmbH–Location Marburg, Baldingerstrasse, D-35043 Marburg, Germany; 2 Coordinating Centre for Clinical Trials-KKS, Philipps-University Marburg, Karl-von-Frisch-Strasse 4, D-35043 Marburg, Germany

**Keywords:** Elective abdominal surgery, Intracutaneous suture, Skin stapling, Superficial surgical site infection

## Abstract

**Background:**

Surgical site infections are the third most frequent type of nosocomial infections. Evidence-based recommendations have been given regarding preoperative hospitalization, hygiene and air-conditioning, patient conditions, and wound dressing. However, no general recommendations concerning wound closure exist. Systematic reviews and meta-analyses suppose a benefit of intracutaneous suture compared to skin staples in orthopedic and obstetric surgery. Literature data for skin closure in elective abdominal surgery are still deficient.

**Methods/Design:**

Patients scheduled for any elective abdominal surgery requiring midline or horizontal laparotomy are potentially eligible for the trial. Trial-specific exclusion criteria are date of admission exceeding four days prior to surgery, antibiotic treatment within the past 14 days, any previous midline or horizontal laparotomy in case the procedure requires the same skin incision as before, neurophysiological deficits or severe psychiatric or neurologic diseases that do not allow an informed consent or compliance, and participation in any other interventional trial with interference of intervention and outcome. The trial is created for process innovation within standardized surgical procedures. It is designed as a prospective randomized controlled single center trial in a parallel design including an active comparator and an intervention group. The intervention addresses the closure of skin after the main surgical procedure: intracutaneous suture in the intervention group and transcutaneous skin stapling in the control group. The rate of superficial surgical site infections is defined as the primary endpoint. Secondary endpoints are time for skin closure, satisfaction with the cosmetic outcome 30 days after surgery, prolongation of hospital stay, and duration of sick-leave due to surgical site infections. The primary efficacy analysis follows the intention-to-treat principle. A χ^2^ test will be applied.

**Discussion:**

The trial is expected to balance the shortcomings of the current evidence. It will help to define the gold standard for wound closure in elective abdominal surgery. Patients’ safety and quality of life are assumed to be enhanced. Therapy costs are likely to be reduced and health care optimized.

**Trial registration:**

German Clinical Trials Register (DRKS) DRKS00004542.

## Background

The prevention of surgical site infections is one of the basic issues in the study of any surgical discipline to enhance quality of health care and patient safety. Over the past decades, this attempt led to the constitution of national institutes for disease surveillance and prevention. For diagnostic purposes, an internationally acknowledged classification for surgical site infections has been outlined by the Center of Disease Control (CDC), Atlanta, USA, within a guideline for prevention in 1999. According to their location and source, surgical site infections are categorized by severity and implications. In Germany, CDC-criteria were adopted by the National Institution for Disease Surveillance and Prevention (Robert Koch Institute) and implemented within the National Reference Center for Surveillance of Nosocomial Infections [1]. A national representative prevalence study (NIDEP-1-trial) demonstrated surgical site infections to be the third most frequent type of nosocomial infections with an incidence of 16% [2], affecting 128,000 patients per year [3] and with a prolonged hospital stay ranging between 9.5 to 23.7 days [4]. Besides patient safety and quality of life, postoperative surgical site infections are of economic relevance, since hospital treatment costs increase by 2.8-fold on average [3]. For example, in the USA, the total economic load due to nosocomial infections was calculated to have reached 95 million USD in 1992 [3].

The main source for surgical site infections is the patients’ own flora, which despite evidence-based preoperative antisepsis, still causes endogenous infections with a proportion of 90% compared to 10% of exogenous infections due to contamination by any external agent [5]. In systematic reviews, meta-analyses, and randomized controlled trials, the length of hospitalization, antisepsis of patient’s skin, surgeons’ hands, operation site, depilation, air-conditioning of the operation room, patient’s temperature, and wound drainage and dressing have proven to be influential in wound healing. Subsequently, standards and recommendations have been outlined by national surveillance institutions [6]. However, no general recommendation for the technique of skin closure exists to date. Staples and different types of sutures are used in clinical routine. Alternatively, adhesive stripes and tissue adhesive DERMABOND ProPen® and Op-site® films are especially favored to close small incisions [7,8]. Results of randomized controlled trials assessing the impact of different closure techniques on superficial surgical site infections are conflicting in dependence of body region and surgical procedure. Differences in the bacterial spectrum and number of bacteria are likely to be influential. In orthopedic surgery, a meta-analysis comparing the outcome of sutures versus staples on superficial surgical site infections demonstrated superiority of sutures for knee and hip surgery. However, the authors outlined substantial methodological limitations of the primary data and the need for definite randomized controlled trials [9]. In heart surgery, a systematic review of randomized controlled trials for closure of leg wounds after vein graft harvesting for coronary artery bypass surgery revealed no difference between sutures and staples [10]. In obstetrics, a recent systematic review and meta-analysis demonstrated the superiority of subcuticular sutures compared to staples for skin closure after caesarean sections; there was no significant difference in the cosmetic outcome [11].

Regarding abdominal surgery, five randomized controlled trials dating from 1981 to 1992, assessed the outcome of staples versus sutures for skin closure on superficial surgical site infection, pain, operation time, and cosmetic outcome. Three of the trials compared interrupted mattress sutures to staples [12-14], and two compared intracutaneous sutures versus staples [15,16], including a comparison of different suture materials [16]. While in the Pickford trial [14] the infection rate was significantly lower in favor of staples (6.3% vs. 17%), no significant difference could be demonstrated in the trials of Eldrup [12] and Gatt [13]. The two trials comparing intracutaneous sutures to staples showed no significant difference regarding the incidence of superficial surgical site infection. Moreover, the suture material was proven to be of no impact [16]. All trials, which additionally considered the cosmetic outcome [13,15,16] and closure time, revealed no significant difference for the cosmetic outcome but a significant reduction of the closure time. However, data addressing postoperative pain were conflicting. Two trials demonstrated a significant reduction of postoperative pain in favor of staples [12,15], whilst one trial showed no difference [13]. Although the evidence is usually classified as “Ib” according to the trial design, the methodology is deficient referring to power calculation, calculation of the effect sizes, allocation, and methods for minimizing bias and blinding, as well as calculation of outcomes when using multiple endpoints. The limitations are explicable considering the methodological progress and consolidated standards of conducting and reporting trials within the past two decades. A recent interdisciplinary meta-analysis of randomized controlled trials investigating the impact of staples versus sutures for skin closure of abdominal wounds shares the same limitations [17]. Altogether, infection rates did not differ significantly comparing suture and staples for skin closure and there were no significant differences in the cosmetic outcome; the primary data were derived from obstetrics and gynecology, general surgery, head and neck surgery, vascular surgery, and emergency care [17]. Despite heterogeneity having been found as not significant, the primary data bear substantial methodological limitations.

Most of the trials above had multiple primary endpoints; neither a calculation of the effect size nor power was reported, and suture materials and techniques varied. To outbalance conflicting results and a lack of evidence, prospective randomized controlled trials are needed to define the ideal closure technique for skin incisions in abdominal surgery.

## Methods/Design

A retrospective analysis of in-house data has been done to prepare the calculation of the sample size and power. This trial manuscript was prepared thereafter. The local ethics committee of the Philipps-University of Marburg gave a positive approval on October 2, 2012 (leading ethics committee number 190/12). The trial is registered at the German Clinical Trials Register (DRKS) (trial number DRKS00004542). Financing is granted through an in-house funding program of the Philipps-University Marburg and Rhoen-AG. The preparation of electronic case report forms, database, and data management system is established.

### Eligibility

Patients scheduled for any elective abdominal surgery requiring a midline or horizontal laparotomy are potentially eligible for the present study. Surgical procedures comprise small bowel and large bowel resection, rectal surgery including laparoscopic assisted colorectal resections with Pfannenstiel incision exceeding six centimeters in length, esophageal, gastric, duodenal, and pancreatic surgery, liver resections, open cholecystectomies with or without choledochotomies, gastro-intestinal and intestinal-intestinal bypass anastomosis, bilio-digestive anastomosis, and splenectomies. After a screening visit, patients will be allocated to the trial according to the below mentioned inclusion and exclusion criteria. Additionally, patient specific risk factors such as age, gender, American Society of Anesthesiologists (ASA)-status, body mass index, diabetes, steroid intake, and smoking habits will be recorded. Before enrolment, an informed consent will be obtained from each patient.

### Inclusion criteria

Key inclusion criteria are scheduled for any elective abdominal surgery using midline or horizontal laparotomy, age greater than or equal to 18 years, expectancy of life greater than 12 months, and informed consent.

### Exclusion criteria

Exclusion criteria are date of admission exceeding four days prior to surgery, antibiotic treatment within the past 14 days, any previous midline or horizontal laparotomy if the procedure requires the same skin incision as before, neurophysiological deficits or severe psychiatric or neurologic diseases that do not allow an informed consent or compliance, participation in any other interventional trial with interference of intervention and outcome, and inability to understand patients’ information and informed consent form.

### Intervention and control

The study is designed as a prospective, randomized, controlled, single center clinical trial in a parallel design including an intervention group and an active comparator. The intervention addresses the type of skin closure in an elective abdominal surgery. In the intervention group, skin closure is performed by intracutaneous suture using Monosyn® 4-0. Skin staples are used in the control group. After the closure of the fascia, patients are randomly assigned to an intervention or control group by intraoperative randomization. Thereafter, the length of the incision and the thickness of the subcutaneous tissue at its thickest point is measured and recorded. The subcutaneous wound is prepared for closure according to the local in-house standard: lavage with diluted chloramine (0.05%), suction drain and closure by absorbable inverting interrupted single stitches. The standard is maintained to control potential confounders of superficial surgical site infections. When the intervention is applied, the time to close the skin is measured and recorded. Further, intraoperative complications such as relevant contaminations of the abdominal cavity which require an additional lavage, extensive blood loss exceeding 500 ml, intraoperative cardiovascular complications or resuscitations, and the total operation time (from the skin incision to skin closure) are registered.

Either the responsible surgeon, or the first or second assistant may close the skin. No special surgical expertise is required since skin stapling and intracutaneous suture are basic surgical skills acquired at the very beginning of surgical training or even in medical training. Altogether, 27 surgeons, including the head of the department, consultants, senior and junior registrars, will participate in the study.

### Surgical methods

Both types of skin closure–transcutaneous stapling and intracutaneous suture–are considered safe and standardized techniques in abdominal surgery. The main surgical procedures are not influenced by the trial protocol. After surgery and closure of the abdominal wall and subcutaneous tissue, merely the type of skin closure will be determined through randomization. The trial is designed for process innovation within standardized therapeutic surgical procedures in an elective setting. No new surgical technique is applied.

### Definition of endpoints and outcome measures

#### Primary endpoint

The primary endpoint is the incidence rate of superficial surgical site infections. Superficial surgical site infection is defined as a category A1 infection according to the CDC, as infection of the incision within 30 days after surgery, involving skin and subcutaneous tissue only and meeting one of the following criteria/clinical signs: (I) purulent secretion, cultural proof of bacteria derived from the tissue covering the incision or any secretion from the incision; (II) pain or tenderness, swelling, redness, warmth; (III) opening of the wound by the treating surgeon; and (IV) diagnosis made by the treating medical doctor.

Type A2 infections are defined as infections within 30 days after surgery and most probably linked to the previous surgical procedure, involving fascia and muscle, and meeting one of the following criteria: (I) purulent secretion from the depth of the incision but not from the organ or visceral cavity; (II) spontaneously opened or opened by a surgeon in case of at least one of the following symptoms: fever (>38°C), localized pain, or localized tenderness; (III) abscess or other signs of infection involving deep tissue layers during another surgery revealed by histopathology or radiology; and (IV) diagnosis made by the treating medical doctor.

Type A3 infections imply infections within 30 days after surgery and most probably linked to the previous surgical procedure and involving the visceral cavity addressed within the previous surgery and meeting one of the following criteria: (I) purulent secretion from a drain addressing the organ or cavity on which the previous surgery was performed; (II) cultural proof of bacteria of a septic secret, organ or cavity; (III) abscess or other signs of infection of the organ or visceral cavity within the clinical examination, subsequent surgery, histopathology, or radiology; and (IV) diagnosis made by the treating medical doctor.

Depending on fulfillment of these criteria, the surgical site infection is assigned to one of the three different categories A1–3, or in case of no infection, “none”. The wound is photodocumented by an independent wound manager on days 2, 5, 10, and 30 after surgery. In case of documented A1–3 infections, the subsequent treatment or procedure is additionally recorded. Category A2 or A3 infections which emerge from deeper layer or even the abdominal cavity and which are not linked to an infection of the upper layer of the skin are not attributed to the method of skin closure and thus do not attribute as failures to the statistical analysis of the primary endpoint.

#### Secondary endpoints

Secondary endpoints comprise time of skin closure, satisfaction of the cosmetic outcome 30 days after surgery, prolongation of hospital stay, and inability to work due to surgical site infections. After randomization and closure of the subcutaneous tissue, the time of skin closure is recorded. Within the follow-up visits, the satisfaction of the cosmetic outcome 30 days after surgery is assessed separately by the patient and an independent wound manager on a scale as used within the German school grade system (1?=?best to 6?=?worst). Moreover, the prolongation of hospital stay and employees’ illness due to surgical site infections are recorded.

### Randomization, allocation concealment, and blinding

A random sequence will be generated through computerized random assignment and concealed allocation. Intraoperative randomization will be performed. After closure of the fascia, the anesthetist or the second scrub nurse calls the randomization office to give the patient’s number, surgeon’s name, and ask for allocation. Using the loudspeaker, the surgeon then is de-blinded. The outcome assessment cannot be blinded due to the nature of the study. Therefore, the detection bias is high and cannot be minimized. Skin staples will be visible for the outcome assessors during the follow-up visits from days 1 to 10.

### Sample size

The trial is designed on the basis of a critical appraisal of current evidence. Literature data concerning the evaluation of intracutaneous suture and skin stapling with regard to superficial surgical site infections are insufficient. Retrospective data of the own clinic served as reference for calculating the sample size. The standard procedure for skin closure has changed during the past years, i.e., in 2011, the patients routinely received intracutaneous suture, while in 2009, the patients had routinely received skin stapling. Retrospective analysis of these data showed a lower rate of A1 infections for intracutaneous (4%) than for stapling (14%) closure. To achieve a power of 80%, 128 patients per group are needed to detect this difference when applying a two-sided χ^2^ test with a significance level of 5%. Incorporating a rate of drop-outs of 10%, a total of 285 patients need to be randomized.

### Statistical analysis

The primary hypothesis is that the rate of superficial surgical site infections within 30 days after elective abdominal surgery via midline or horizontal incision is lower in patients after intracutaneous closure of the skin when compared to closure of the skin by staples. The primary efficacy endpoint will be assessed as a rate, defined as the number of patients who experienced a superficial surgical site infection (type A1) within 30 days after surgery divided by the number of all patients randomized to this arm. Infections of types A2 or A3 are not defined as primary events. For the intention-to-treat analysis, these patients will be considered within the denominator. The primary analysis will be done in the intent-to-treat population. To compare the incidence rates in both arms, a two-sided χ^2^ test will be applied. As sensitivity analyses, a χ^2^ test for the per-protocol population will be applied as well as logistic regression analyses adjusting for potential prognostic factors: diagnosis and procedure, age, gender, ASA-status, body mass index, diabetes, steroid intake, smoking, and thickness of subcutaneous fat. The secondary efficacy endpoints will be analyzed exploratively. Appropriate summary measures for the empirical distributions as well as two-sided 95% confidence intervals and *P* values will be reported. The safety analysis will be applied to the safety population comprising all patients operated.

## Discussion

Surgical site infections are the third most common type of nosocomial infections. National health surveillance systems give evidence-based recommendations for their prevention and treatment. However, no general recommendation can be given for the closure of skin in elective abdominal surgery. To date, literature data are deficient. A retrospective in-house data analysis and a systematic review and meta-analysis for skin closure after cesarean sections in obstetrics lead to the conclusion that intracutaneous skin closure may reduce the incidence of superficial surgical site infections. A prospective randomized clinical trial will help to define the gold standard for skin closure in elective abdominal surgery. A lower incidence of superficial surgical site infections will reduce nosocomial infections, enhance the quality of health care and patient safety, and will be of economic relevance.

## Trial status

Enrolment started on 4^th^ March 2013.

## Abbreviations

ASA: American Society of Anesthesiologists; CDC: Centers for Disease Control and Prevention.

## Competing interests

The authors hereby declare that there are no financial or non-financial competing interests neither within the conception nor conduction of the trial.

## Authors’ contributions

DKB queried if intracutaneous wound closure in elective abdominal surgery may reduce the incidence of superficial surgical site infections. He reviewed the trial protocol and contributed substantially to the trial concept. KM and LM conducted a systematic Pubmed literature research on the current evidence and generated the hypothesis. They did a retrospective analysis on in-house data to prepare the calculation of sample size and power. They worked out a trial concept and created a study protocol. They applied for an ethic vote, in-house funding, registered the study, and wrote the present paper for TRIALS publication. CH and AR carried out all statistical calculations and outlined the biometric concept, which was reviewed by CSB. For diverse documents (proposal for ethics committee, application for funding, and this manuscript) they wrote the statistical paragraphs and also contributed to the successful submissions through holistic reviewing prior to submission. All authors read and approved the final manuscript.
